# Hypokalemic Paralysis Is Not Always Periodic: A Case Series

**DOI:** 10.1155/carm/9925534

**Published:** 2025-08-25

**Authors:** Mohak Jain, Gaurav Mehta, Ajay Parmar, Minal Shastri, Sahaj Patel, Vidhi Gandhi, Shifa Karatela, Apurva Patel

**Affiliations:** Department of Medicine, Medical College Baroda, Vadodara, Gujarat, India

**Keywords:** channelopathy, hypokalemic paralysis, periodic paralysis, renal tubular acidosis, thyrotoxic periodic paralysis

## Abstract

Potassium is vital for cellular function, particularly in excitable tissues like nerves and muscles, which rely on potassium gradients to function normally. Hypokalemia can lead to severe issues such as muscle weakness and irregular heart rhythms. This case series presents four instances of hypokalemic paralysis, a neuromuscular condition that can be either periodic or isolated. The cases vary in presentation and treatment approach, highlighting the need to differentiate between primary and secondary causes of hypokalemia for optimal management. Fast and accurate diagnosis through laboratory tests and history taking is critical, and while acute management is standard, preventive strategies depend on the underlying cause. Further research is needed to establish definitive guidelines for the prevention of hypokalemic paralysis.

**Summary**

• Early diagnosis of hypokalemic paralysis through lab tests and ECG is crucial to prevent life-threatening complications such as respiratory failure.
• It is also important to identify the etiology of hypokalemic paralysis. While the primary is genetic, secondary can be due to thyrotoxicosis, renal tubular acidosis, or GI losses to tailor long-term preventive therapy.

## 1. Introduction

Potassium gradient across cell membrane is essential for proper cellular function of nerves and muscles. Imbalances in potassium levels, whether low or high, can lead to severe problems, including irregular heart rhythms and muscle weakness. These imbalances often result from issues with how the kidneys manage potassium, which can be affected by systemic diseases [1].

Hypokalemia can present with cardiovascular, neuromuscular, or gastrointestinal manifestations. Paralysis secondary to hypokalemia can be periodic paralysis (PP) or just independent episode [2]. Hypokalemic periodic paralysis (HPP) is the neuromuscular manifestation, forming one of the four disorders under the PP umbrella. The other disorders are hyperkalemic PP, thyrotoxic PP, and Andersen–Tawil syndrome [3]. The symptoms of different hypokalemic paralysis remain the same. However, the management is adjusted according to the etiology and other comorbidities. We present a series of four cases of hypokalemic paralysis, each with a unique presentation, etiology, and treatment.

## 2. Cases

### 2.1. HPP

A 35-year-old male presented with complaints acute onset weakness in all four limbs. The patient denied any abdominal pain, back pain, diarrhea, sensory abnormalities, or neurological deficits. He reported a history of similar episodes: one which was 2 years ago of the same severity, and another 6 months ago, which was less severe. Both episodes resolved spontaneously within a day. There was no significant family history.

On general examination, the patient was bradycardic. Muscle tone was decreased, and power in all four limbs was decreased, graded at 1. Superficial reflexes were intact, while deep tendon reflexes were +1, indicating hyporeflexia. The remainder of the central nervous system (CNS) examination was unremarkable.

Laboratory investigations revealed hyponatremia (Serum Sodium = 128 mEq/L) and hypokalemia (Serum Potassium = 2.3 mEq/L).

The patient was administered intravenous potassium chloride (KCl) at 20 mEq/hour (total 120 mEq of KCl) with electrocardiography (ECG) monitoring along with oral correction. Further genetic testing identified sodium voltage-gated channel alpha subunit 4 (SCN4A) mutation in the patient.

### 2.2. Thyrotoxic Hypokalemic Paralysis

A 35-year-old male presented to the emergency room with weakness of all four limbs for the past 6 h. Initially, he found himself unable to rise from a chair or walk. Within an hour, the weakness had progressed to his upper limbs, manifesting as an inability to raise his arms above shoulder level and difficulty holding objects. The weakness was acute in onset, progressive, and symmetrical.

There was no history suggestive of sensory involvement, bowel or bladder dysfunction, trauma, muscle pain, fever, diarrhea, vomiting, abdominal pain, consumption of canned foods, unknown bites, significant drug use, or ingestion of toxic compounds. There was no previous history of similar complaints in the patient or his family, and the patient did not have any known chronic medical conditions. His personal history revealed occasional alcohol consumption (once every fortnight). The patient was initially treated at a primary health care facility, where he received intravenous fluids, following which his weakness worsened. Subsequently, the patient became tachypneic with declining oxygen saturation, necessitating urgent intubation. He was transferred to the intensive care unit due to ascending paralysis involving the respiratory muscles.

A general physical examination on admission was unremarkable, and nutritional assessment was normal. On CNS examination, the patient exhibited decreased muscle tone, areflexia in all four limbs, and a muscle power grade of 1 across all limb joint movements.

Investigations revealed severe hypokalemia (serum potassium 1.6 mEq/L) secondary to thyrotoxicosis due to Graves' disease, with decreased thyroid-stimulating hormone (TSH) and elevated triiodothyronine (T3) and thyroxine (T4) levels. TSH receptor antibody levels were elevated at 12.54 IU/L (normal value < 1.22 IU/L). ECG showed T-wave inversion in lateral leads along with QTc prolongation.

The patient was administered intravenous potassium at 20 mEq/hr (100 mEq of KCl was administered), followed by enteral supplementation resulting in significant improvement in his weakness, and he was extubated within three hours of correction. Supportive treatment was provided. Further history revealed that the patient had experienced hand tremors, weight loss, and excessive sweating for the past 1.5 months. He was started on carbimazole (10 mg every 12 h) to manage Graves' disease and propranolol (20 mg every 12 h) to control adrenergic symptoms, with a plan to reduce the carbimazole dose to once daily upon achieving a euthyroid state.

### 2.3. Hypokalemic Paralysis Secondary to Renal Tubular Acidosis (RTA)

A 55-year-old male experienced acute onset generalized weakness on waking up. He was unable to move in bed, raise his arms, or hold his head up. After admission, he developed breathing difficulties and drowsiness, requiring intubation and one day of mechanical ventilation.

The patient denied any tingling or numbness in his limbs, neck pain, bladder disturbances, headaches, seizures, dysphagia, or diplopia. He also denied history of ptosis, recent trauma, diarrhea, vomiting, unknown bites, or recent vaccinations.

The patient had a history of similar complaints in 2018. In the month of May, he was admitted to a municipal hospital in Surat with sudden onset weakness in all four limbs. He was immediately intubated and required ventilator support for 2 days. Investigations at that time revealed hypokalemia, which persisted on repeated testing. He was subsequently discharged after a 10-day hospital stay with a prescription for acetazolamide 250 mg twice daily and syrup potassium chloride (KCl). He took the medication for 1 year before discontinuing it of his own volition.

On CNS examination, the patient exhibited hypotonia in all four limbs, with zero power in all four limbs. Deep tendon reflexes were diminished, while superficial reflexes were intact. Sensory function was also preserved.

On the day of admission, investigations revealed a serum potassium level of 2.5 mEq/L and arterial blood gas analysis (ABGA) suggestive of standard anion gap metabolic acidosis. Urinary pH was 7.5 while urinary potassium, sodium, and chloride were normal. An electrocardiogram showed T-wave inversion in leads V1 to V6. The urine protein-creatinine ratio (UPCR) was elevated.

Based on the patient's history, examination findings, and investigations, the probable diagnosis was secondary hypokalemic paralysis due to idiopathic distal RTA type 1.

The patient was treated with intravenous potassium chloride (KCl) at 20 mEq/hour. This was followed by oral potassium citrate (alkaline agent) syrup, 20 mL three times daily, diluted. After symptomatic improvement, the patient was advised to continue oral potassium citrate tablets (10 mEq), one tablet three times daily, along with a potassium-rich diet and avoidance of a high-carbohydrate diet.

### 2.4. Hypokalemic Paralysis Secondary to GI Loss

A 33-year-old male with no significant past medical history presented to our emergency department with sudden onset weakness in all four limbs for one day and history of diarrhea for the past week. The patient had been relatively asymptomatic until 1 week prior, when he developed diarrhea, characterized by 5-6 watery episodes per day. The diarrhea was not associated with fever, abdominal pain, nausea, or vomiting and resolved over the next few days. However, on the day of presentation, on waking up to reach for a glass of water, he noticed that he could not get out of bed or raise his arms, though he could turn in bed. The patient developed acute onset generalized weakness.

The patient reported no neck pain, backache, bladder disturbances, symptoms suggestive of sensory involvement, headaches, dysphagia, diplopia, recent drug intake, unknown bites, or recent vaccinations.

On CNS examination, the patient had decreased tone in all 4 limbs. Reflexes were present albeit weak. Muscle strength was graded 3/5 in the upper limbs and 2/5 in the lower limbs.

Laboratory investigations revealed hypokalemia with a serum potassium level of 2.9 mEq/L, while the remaining lab results were unremarkable. Serum Mg was borderline normal (1.5 mEq/L). The patient was treated with intravenous potassium chloride (KCl), administered at a rate of 20 mEq/hour (total quantity infused: 80 mEq). This resulted in rapid resolution his symptoms, and the patient was subsequently discharged.

## 3. Results

Table 1 shows the ECG findings, etiology, and treatment history of study subjects.

## 4. Discussion

Table 1 summarizes all four cases and their brief presentations of hypokalemic paralysis. Hypokalemic paralysis classically presents with quadriparesis but can present with an atypical presentation like hemiparesis or isolated muscle involvement [2]. Hypokalemic paralysis can be primary or secondary, depending upon the underlying cause. Defects in calcium and potassium channels cause primary hypokalemic paralysis, while secondary hypokalemic paralysis is due to underlying systemic issues. Our case series address both kinds of cases [4].

PP, which presents as episodes of muscle weakness caused by several precipitating factors, is considered HPP when associated with low potassium levels. HPP is the most common cause of PP, yet it is rare. Epidemiologically, the global prevalence is estimated to be 1 in 100,000 [5]. The gender distribution lies more toward males, with men affected three to four times more than women [5]. HPP can be inherited or acquired with other disorders like thyrotoxicosis. Hypokalemic and hyperkalemic, both PPs, have autosomal dominant inheritance and are usually present in adolescence [6]. Contrary to common presentation, our case developed complaints in adulthood, which can be attributed to some cases of missed diagnosis or neglect of mild symptoms.

The pathophysiology for familial or inherited HPP stems from the genetic mutation in calcium or sodium channels, with some cases of unidentified mutations. There are two forms of familial HPP depending on the involved channel: type 1, which is more common, includes dihydropyridine-sensitive skeletal muscle calcium channel gene mutation (*CACNA1S*), while type 2, on the other pole, involves voltage-sensitive skeletal muscle sodium channel gene mutation (*SCN4A*). There have been cases of mutations affecting potassium rectifier channels, and some instances have unidentified mutations; they form the less common group [5]. Acquired causes, as mentioned, have been reported associated with hyperthyroidism, which was found in one of our cases.

The differential diagnosis involves other causes of PP and causes of muscle weakness secondary to hypokalemia. Other causes of PP are differentiated based on the presence or absence of associated features, such as hyperkalemia in hyperkalemic PP, symptoms, and laboratory manifestation of hyperthyroidism in thyrotoxic PP, and the presence of dysmorphic features and arrhythmias in Anderson-Tawil syndrome [7]. The causes that present as muscle weakness are secondary to hypokalemia and are usually due to renal, gastrointestinal, or some other underlying systemic issues [8].

Apart from the causes mentioned above, there are other causes of paralysis, such as myasthenia gravis, paramyotonia congenita, Guillain–Barre syndrome, spinal cord syndromes, stroke, and neuromuscular junction disorders [6]. However, the presence of hypokalemia would direct the physician to mentioned differentials of paralysis.

Patients of HPP and paralysis secondary to hypokalemia would present with the same features, and the differentiation relies upon detailed history and genetic evaluation in suspected HPP cases. All four patients presented with complaints of weakness in all four limbs, and their initial lab investigations revealed hypokalemia. Other initial assessments should focus on ECG, thyroid panel, blood gas analysis, and other electrolyte evaluation [7]. The presence of hypokalemia between the attacks helps separate HPP vs. paralysis secondary to hypokalemia. Several case studies have demonstrated that initial lab work along with subtle clinical clues can help in differentiating distal RTA from HPP [8, 9].

Managing all the causes would follow similar acute treatment but different preventive measures. As per the protocol, 30 mEq of potassium should be given every 2 hours with a maximum of 90 mEq per 24 h. Some studies also suggest an even slower potassium supplement of 10 mEq orally [10]. A study reported 80% of cases of rebound hyperkalemia in patients who were given more than 90 mEq in the first 24 h [11]. Rebound hyperkalemia can be avoided by confirming hypokalemia before the initiation and controlling the potassium dose administered. Other considerations that should be considered are cardiac monitoring and avoiding administration with dextrose, as the insulin secretion would impair the potassium levels [7].

Paralysis attacks can be prevented by avoiding the precipitating factors or treating the underlying cause. In the case of thyrotoxic PP, antithyroid drugs are recommended for prevention. For HPP, chronic potassium supplementation has been used apart from lifestyle changes. Studies have reported the effectiveness of certain medications like carbonic anhydrase inhibitors, potassium-sparing diuretics, calcium channel blockers, and potassium channel openers. More research is required in this field to reach a definitive treatment [12, 13].

Thus, based on these cases, it can be concluded that the causes of paralysis in cases of patients with hypokalemia should be differentiated. Fast, efficient laboratory investigations would help in guiding optimal treatment. While there are no definitive guidelines for prevention, decisions should be made on a case-to-case basis.

## Figures and Tables

**Table 1:** Clinical presentation and treatment history of study subjects.

Serial no.	Age	ECG (key findings)	Cause	Active treatment	Preventive treatment
1.	35	Prolonged PR, U waves present in V1 to V3	Primary	IV KCl	Take a high potassium diet, avoid high carbohydrate diets
2.	35	ST depression in leads I and aVL. T-wave inversion in leads I, aVL, V4, V5, V6	Secondary to thyrotoxicosis	IV KCl	Carbimazole, propranolol
3.	55	T-wave inversion in leads V1 to V6	Secondary to renal tubular acidosis	IV KCl	Take a high potassium diet, potassium supplementation, alkali therapy
4.	33	Prominent U waves in precordial leads	Secondary to gastrointestinal loss	IV KCl	Not needed

## Data Availability

The data that support the findings of this study are not openly available due to reasons of patient confidentiality. Further inquiries can be directed to the corresponding author.
